# Making “Good” Choices: Social Isolation in Mice Exacerbates the Effects of Chronic Stress on Decision Making

**DOI:** 10.3389/fnbeh.2020.00081

**Published:** 2020-05-25

**Authors:** Arish Mudra Rakshasa, Michelle T. Tong

**Affiliations:** ^1^Neuroscience Program, Earlham College, Richmond, IN, United States; ^2^Biochemistry Program, Earlham College, Richmond, IN, United States; ^3^Department of Psychology, Earlham College, Richmond, IN, United States; ^4^Neuroscience Program and Department of Biology, University of St. Thomas, St. Paul, MN, United States

**Keywords:** cost-benefit conflict, chronic stress, repeated immobilization, social isolation, decision-making, adaptive behavior, stress reaction

## Abstract

Chronic stress can impact decision-making and lead to a preference for immediate rewards rather than long-term payoffs. Factors that may influence these effects of chronic stress on decision-making are under-explored. Here we used a mouse model to investigate the changes in decision-making caused by the experience of chronic stress and the role of social isolation in exaggerating these changes. To test decision-making, mice were trained to perform a Cost-Benefit Conflict (CBC) task on a T-maze, in which they could choose between a high-reward, high-risk alternative and a low-reward, low-risk alternative. Mice were either housed in groups or alone throughout the experiment. Both groups of mice underwent a seven-day period of repeated immobilization to induce chronic stress. Stress levels were confirmed using behavioral (open field test) and physiological (urine corticosterone ELISA) measures. We found a significant increase in frequency of high-risk decisions after exposure to chronic stress among both socially- and individually-housed mice. Crucially, socially-housed mice showed a significantly smaller increase in high-risk decision-making compared to singly-housed mice. These findings suggest that chronic stress leads to an increase in high-risk decision-making in mice, and that lack of social interaction may exacerbate this stress effect.

## 1. Introduction

“Poverty to a large extent is also a state of mind,” said Housing and Urban Development Secretary Ben Carson, a top US government official tasked with increasing access to affordable housing, in a radio interview for National Public Radio (Fessler, 2017). Secretary Carson's comments reflect a widely held belief that poverty is caused by individual decision-making, where people living in poverty are stereotyped as making bad choices like buying iPhones rather than investing in healthcare (Bump, 2017). Such comments suggest that people living in poverty make decisions based on inherently flawed values, and would escape the harsh conditions of poverty if only they changed their “state of mind.” Put another way, poor decision-making causes poverty.

This commonly-held view, however, does not account for the effects of poverty-related chronic stress on individuals' physical and mental health. Defined as prolonged and constant exposure to negative emotional experiences, chronic stress has been linked to negative health outcomes such as increased incidences of coronary heart disease (Vitaliano et al., 2002), the acceleration of memory impairments (Jeong et al., 2006), and increased tumor growth (Thaker et al., 2006). In addition, prolonged exposure to stressful environments may be correlated with increased vulnerability to mental health issues such as depression (Pearlin et al., 1981; Cohen et al., 2007) and substance abuse disorders (Brady and Sinha, 2005; Sinha, 2008). Other mental health disorders, including post-traumatic stress disorder (PTSD) (Baum and Posluszny, 1999) and schizophrenia (Walker and Diforio, 1997), may also be triggered or aggravated by chronic stress. Therefore, chronic stress has well-documented negative effects on both physical and mental health of individuals.

Of particular relevance to this study, chronic stress has been shown to affect cognitive processes such as decision-making (Janis and Mann, 1976; Ceccato et al., 2016). More specifically, persistent exposure to stressful situations can lead to increased risk-taking (Zur and Breznitz, 1981). For instance, military students under severe chronic stress were more likely to fire at targets even when they found that the targets were humans and not humanoid dummies (Larsen, 2001). Chronic stress can also lead to changes in decision-making about personal health, causing individuals to prefer high, immediate rewards rather than delayed payoffs in both food choice (Oliver et al., 2000) and substance use (Fishbein et al., 2006). Mercer and Holder (1997) found that chronically-stressed individuals were more likely to choose “comfort foods” over healthy food. These studies suggest a strong relationship between chronic stress and an increased preference for immediate rewards in decision-making.

A direct causal relationship between chronic stress and decision-making, however, can be challenging to establish for human participants, since human studies necessarily rely on self-reports from participants who are already chronically stressed. In this regard, animal models can provide an effective means of studying the effects of chronic stress on decision-making. Chronic stress may be reliably induced in rodents using several well-established stress protocols (Marin et al., 2007; Monteiro et al., 2015). As such, researchers can manipulate physiological and environmental conditions to explore causality of chronic stress effects and assess these effects at different time points. Animal models also allow researchers to better understand biochemical and neural effects of chronic stress. Moreover, rodents that are modified to mimic certain physiological or psychological conditions relevant to humans may provide insight into stress-related responses in humans (Sutanto and Kloet, 1994).

Several studies of chronic stress with rodent models (Pham et al., 2013; Zeeb et al., 2013) have shown that mice exposed to chronic stressors exhibit decreased evaluation of choices and reduced hesitation in making a decision (Pardon et al., 2000). Rats subjected to such chronic stress make decisions based on habit rather than an evaluation of consequence (Dias-Ferreira et al., 2009). Friedman et al. (2017) have recently demonstrated that mice engage in “aberrant” decision-making behavior after being subjected to chronic immobilization stress. When the researchers presented mice with the decision to choose between a high-reward, high-risk alternative and a low-reward, low-risk alternative, they found that chronically-stressed mice “were significantly more likely to choose high-risk/high-reward options than matched controls” (Friedman et al., 2017, p. 1,192), indicating that mice exposed to chronic stress preferred immediate high payoffs in their decisions, whereas unstressed mice consistently preferred low-risk alternatives. These studies indicate that chronic stress has a significant influence on decision-making, such that it may actually be said to cause individuals to favor high-risk, high-reward decisions. Thus, we recommend a shift from framing high-risk decisions as “aberrant” or “bad,” and suggest, instead, that choosing risky immediate rewards may in fact be “adaptive” in stressful, unpredictable environments.

These results have strong implications for our understanding of human decision-making. They suggest that chronic stress causes a cognitive bias toward high-risk, high-reward choices, which may at times perpetuate conditions of chronic stress. Thus, it is critical to explore factors that may counteract these cyclical effects. There is some evidence that social interaction could attenuate the effects of chronic stress (DeVries et al., 2003). Regular, healthy social interaction may create a buffer against chronic stress (Cohen and Wills, 1985), such that the effects of chronic stress may be mediated by the access to social support offered by one's living conditions (Quittner et al., 1990; Esterling et al., 1994). People who do not have access to healthy social interaction, such as older adults living in isolation, seem to face increased stress and risk of cardiovascular disease (Xia and Li, 2018). Conversely, older adults who receive emotional support from social settings such as churches report lesser effect of financial stressors on self-rated health (Krause, 2006). Thus, housing conditions and availability of healthy social support may be significant in mitigating the effects of chronic stress.

Given that mice and rats are also social animals, and that responses such as pain sensitivity are modulated by social factors in mice (Langford et al., 2006), housing conditions among rodent models offer an operationalization of social support in humans. Social housing was shown to lessen the effects of chronic stress on behaviors such as sexual activity in rats, whereas rats raised in isolation were more vulnerable to chronic stress-induced behavioral changes such as decreased sexual activity (Paolo et al., 1994). Rats housed socially performed much better on spatial memory tests than those raised in isolation (Nilsson et al., 1999), suggesting that social interaction may improve or preserve basic cognitive functions. Additionally, Berry et al. (2012) have shown that being subjected to social deprivation represents a stressful condition for mice, and that mice in different housing conditions may use different adaptive responses to these stressors. Notably, social housing in male rats particularly seems to reduce the effects of chronic stress and prevent significant decreases in neurogenesis (Westenbroek et al., 2004), which may suggest that housing condition may interact significantly with the effects of chronic stress on cognitive functions such as decision-making. Thus, social interaction may be a potent factor in attenuating the effects of chronic stress in both animals and humans.

The current study investigated the possible role of social isolation in exacerbating stress-induced changes in decision-making in mice. Although the effects of chronic stress and social isolation on decision-making have separately been studied, to our knowledge, the interaction between these three factors has not yet been explored together. Decision-making and stress were assessed in socially-housed and singly-housed mice using behavioral and physiological measures, with adaptive decision-making defined as a greater tendency to choose a high-reward alternative in conditions of stress. There were two research hypotheses for the current study: (1) Chronic-stress through repeated immobilization would cause increased high-risk/high-reward decision-making in all mice, and (2) social isolation would exaggerate the effect of chronic stress on decision-making, such that socially-housed mice would show a smaller increase in high-risk/high-reward decisions than singly-housed mice after chronic stress.

## 2. Methods

### 2.1. Animals

Thirty male C57BL/6NCrl mice (Charles River Laboratories, 7–8 weeks old at the time of arrival) were used in this experiment. Immediately after arrival at the housing facilities, the mice were randomly assigned to home cages alone (singly-housed, *n* = 15) or in groups of five (socially-housed, *n* = 15). Animals were kept on a 12:12 h reverse light/dark cycle for the duration of the experiment. All mice were housed at constant temperature (70°F) in standard Plexiglass cages (28 × 17 × 12 cm) and were given unrestricted access to food and water throughout the study. All procedures performed with rodents were approved by the Institutional Animal Care and Use Committee at Earlham College prior to the start of the study and were conducted in accordance with the U.S. National Research Council Guide for the Care and Use of Laboratory Animals.

### 2.2. Procedure

This study employed a Pre-Post-Control mixed-measures design to compare effects of chronic stress on decision-making between socially-housed and isolated mice. Briefly, all mice underwent an initial Open Field Test (behavioral measure of stress), urine collection for corticosterone ELISA assays (physiological marker of stress), and the Cost-Benefit Conflict task (measure of decision-making). Following these pre-stress tests, all mice were subjected to seven days of repeated immobilization to induce chronic stress. Finally, mice were tested again using the open field test, urine corticosterone ELISA assay, and the CBC task (no pre-training trials during the post-stress test) to measure post-stress levels of stress and decision-making.

#### 2.2.1. Open Field Test

All mice were tested in the Open Field Test twice, before and after the chronic stress manipulation. The Open Field Test is a canonical behavioral test commonly used in rodent research as a measure of anxiety-like behavior that is sensitive to stress, based on the natural tendency of mice to exhibit thigmotaxis (remaining close to the periphery or walls of a novel surrounding) when stressed (Gould et al., 2009). A testing arena (Plexiglass, 28 × 17 × 12 cm) was divided into a circular “Central Zone” (radius 6.35 cm, area = 127 cm^2^) and “Exterior Zones” (remaining area = 349 cm^2^). The Exterior Zone comprises a larger area than the Central Zone, but this is standard in protocols for the Open Field Test (Seibenhener and Wooten, 2015), and still allows for a reliable behavioral assessment of stress. Mice were placed in the center of the arena and the total time spent with all four feet within the Central Zone over a 5-minute session was recorded manually using a stopwatch.

#### 2.2.2. Urine Corticosterone ELISA

An elevation in corticosterone levels is a natural and reliable response to the experience of stress in mice (Apfelbach et al., 2005). Previous studies have used urine corticosterone ELISAs to measure stress in mice (Van der Meer et al., 2004; Mesa-Gresa et al., 2016). Urine samples were collected from mice using Fitchett's method (Fitchett et al., 2005) immediately following the Open Field Test during both the pre-stress and post-stress sessions. Mice were transferred to an empty Plexiglass cage similar to their regular housing cages and allowed to roam freely. Samples were collected using a micropipette after the mice urinated and pooled for each mouse to collect approximately 100 μL of urine for each mouse. Samples were stored at −80°C immediately after collection.

To measure corticosterone levels, the samples were thawed to room temperature and diluted 20-fold with SBS diluent as per the assay manufacturer's recommendation. ELISA assays were only performed for a subset of mice (*n* = 20) in the study—10 socially-housed mice and 10 singly-housed mice. Corticosterone concentrations in the urine samples were determined using corticosterone ELISA kits according to manufacturer's instructions (Corticosterone ELISA Kit, AssayPro LLC, St. Charles, MO), with triplicates for each urine sample. Absorbances for samples were measured at 450 nm using the SpectraMax 190 Microplate Reader (Molecular Devices, San Jose, CA). Since the urine corticosterone ELISA is a competitive ELISA, absorbances were transformed into % Bound (*B*/*B*_0_) for all samples using Maximum Binding (*B*_0_) controls. Urine corticosterone concentrations (ng/mL) were determined from these transformed absorbances using 4-Parameter Logistic Regression for standard curve-fitting. Transformations and curve fitting were conducted using a web-based ELISA analysis tool at www.elisaanalysis.com.

#### 2.2.3. Cost-Benefit Conflict (CBC) Task

##### 2.2.3.1. Pretraining trials

Pre-training lasted for 5 days, during which time all mice were already separated into singly-housed and socially-housed groups. All mice were habituated to the experimenter and to the T-maze apparatus (Maze Engineers, Boston, MA; stem 30 × 10 cm, arm length 30 cm each) used for the CBC test at the beginning of each pre-training day. Mice were simply allowed to roam on the T-maze for two minutes. No stimuli or reward were present at this time. Following this habituation period, each maze arm was fitted with a food well (1 cm in diameter) and a clip-on lamp at the far end. Chocolate whole milk purchased from a local grocery store, either pure (high reward) or diluted with water to an 80% dilution by volume (low reward), was used as a reward for mice in the food wells. All mice were trained in receiving the chocolate milk reward from the food wells in the T-maze using 10 repeated forced-choice trials. The lights were *not* turned on during pre-training.

##### 2.2.3.2. Training trials

**Figure 1:**
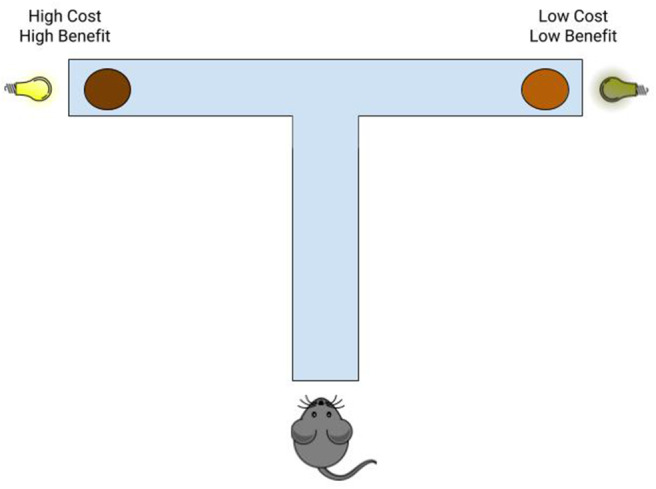
Schematic representation of the Cost-Benefit Conflict (CBC) task. A T-maze was used to assess decision-making in mice using the CBC task, in which mice chose between a high-cost/high-reward alternative (pure chocolate milk paired with bright light) and a low-cost/low-reward alternative (dilute chocolate milk paired with dim light).

Each mouse underwent ten forced-choice training just prior to experimental trials. The food reward alternatives during Training were the same as pre-training, pure chocolate milk (a high reward) in one arm and a dilute (80% v/v) chocolate milk (low reward). For Training, however, the lights were turned on. We paired the high reward with a very bright (1,600 lumens) light (a high cost) and low reward with a dim (100 lumens) light (low cost; Figure 1). The use of high-intensity light as an aversive stimulus is based in the natural aversion of mice to bright lights. The use of light/dark transition test as a validated measure for anxiety-like behaviors in mice (Serchov et al., 2016) as well as extensive cost aversion testing conducted by Friedman et al. (2017) demonstrate that even highly inbred laboratory mice show significant aversion to bright light, as wild mice are expected to do in natural settings. One arm of the maze was blocked off using a guillotine door, so that the mice were forced to explore the only open arm. The arm that was open to explore was alternated between each forced-choice training trial.

##### 2.2.3.3. Experimental trials

Finally, mice were given 20 free-choice experimental trials. The set-up was identical to Training Trials; however mice were released onto the stem of the T-maze with both arms of the maze open for exploration. A mouse was considered to have made a “decision” when it drank at least some chocolate milk from the food well of an arm. No decision was recorded if a mouse chose not to drink from either food well after 2 min on the maze. Data from the 20 experimental trials for each mouse were averaged to find % of decisions in which a mouse chose the high-risk/high-reward alternative.

#### 2.2.4. Repeated Immobilization Protocol

Mice were immobilized for 1 h each day for 7 consecutive days. They were immobilized using mouse restraint bags (Animal Identification and Marking Systems, Inc., Catalog Number 89066-344). These restraint bags were small enough to significantly limit the animals' mobility but large enough for the animals to breathe freely. Mice were placed on a flat surface and coaxed into entering the wide end of the bags, after which the tail end was tied with a zip-tie to prevent escape. Mice that were immobilized in this manner were placed on a flat surface and monitored regularly to ensure free breathing.

### 2.3. Statistical Analysis

Our study was a 2 (Housing Condition: Single or Group) × 2 (Stress Exposure: Pre- and Post-) mixed design. We had three separate dependent measures: urine corticosterone concentration, time spent in the center of the open field, and % of high risk/high reward decisions. We performed a linear mixed effects analysis for each of the three measures using R 3.6.1. The fixed effects were Housing Condition and Stress Exposure, and to account for intrinsic performance differences between mice, all analyses also included a random effect of mouse. Our main hypothesis for all three dependent measures is that animals in the two housing conditions would respond differentially to restraint stress which would be supported by a significant interaction in the omnibus F test rather than any single pairwise comparison. However, for the sake of thoroughness, we used estimated marginal means to perform *post-hoc* tests and corrected for multiple comparisons using Tukey's method. The results of these comparisons are provided in Table S2.

## 3. Results

### 3.1. Repeated Immobilization Reliably Induced Stress, but Social Housing Decreased Stress-Induced Anxiety in the Open Field Test

Data from the Open Field Tests and urine corticosterone ELISA assays were analyzed to determine whether repeated immobilization was successful in inducing chronic stress in the mice and whether housing condition affected stress measures. For both the Open Field and ELISA data, we ran a linear mixed effects model with two factors: Housing Condition (Singly or Socially) and Immobilization (Pre- and Post-Immobilization), with random effect of mouse.

#### 3.1.1. Open Field Tests

**Figure 2:**
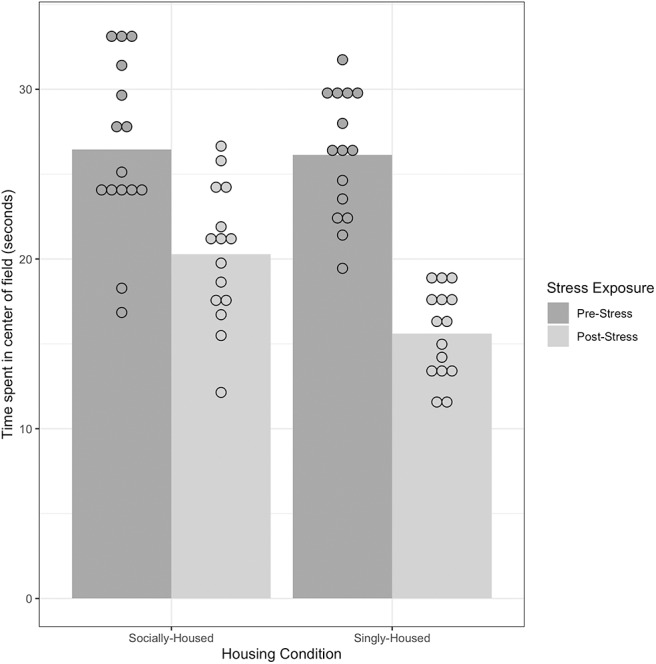
Performance of socially-housed (*n* = 15) and singly-housed mice (*n* = 15) on an Open Field Test before and after exposure to chronic stress. A decrease in mean time spent in the center of the open field indicates an increase in behavioral stress levels. There was a significant interaction of Housing Condition and Stress Exposure, *F*_(1, 30)_ = 13.44, *p* < 0.001, $\eta_{p}^{2}$ = 0.31, indicating that singly-housed mice showed a greater decrease in open field activity after exposure to chronic stress than socially-housed mice. Dots represent individual data points in each group, bars represent the mean for each group. See Supplementary Materials for pairwise comparisons.

The mean time spent in the center of the open field (“open field activity”) was the dependent measure, where greater open field activity would indicate lower stress levels. The results (Figure 2) showed a significant main effect of Immobilization with a large effect size, *F*_(1, 30)_ = 197.50, *p* < 0.001, $\eta_{p}^{2}$ = 0.87, suggesting that the stress protocol was effective. There was no significant main effect of Housing Condition, *F*_(1, 30)_ = 3.94, *p* = 0.06, $\eta_{p}^{2}$ = 0.12. There was also a significant interaction of Immobilization and Housing Condition, *F*_(1, 30)_ = 13.44, *p* < 0.001, $\eta_{p}^{2}$ = 0.31, such that singly-housed mice showed a greater decrease in open field activity after repeated immobilization than socially-housed mice (see Supplementary Materials for group summary statistics and pairwise comparisons). The results show that our stress protocol was effective in inducing stress, and that it produced a greater change among singly-housed mice than socially-housed mice on a canonical behavioral test of stress.

#### 3.1.2. Urine Corticosterone

**Figure 3:**
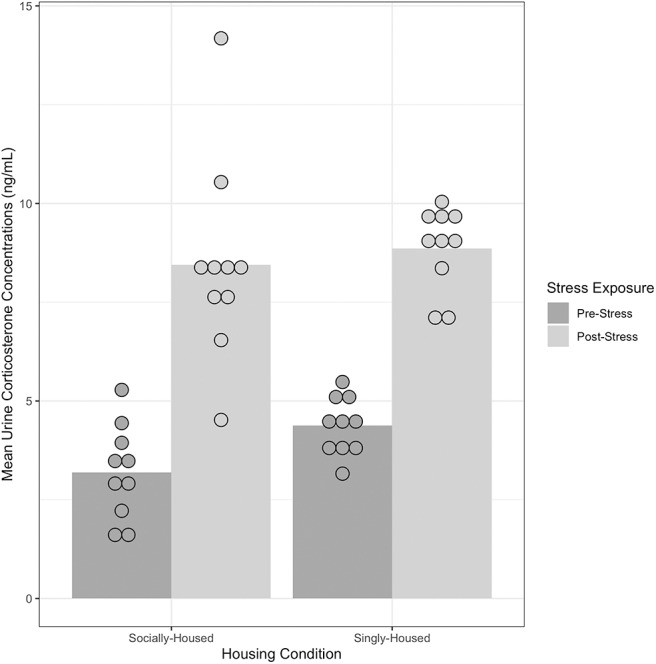
Urine corticosterone concentrations of socially-housed (*n* = 10) and singly-housed mice (*n* = 10) measured using urine corticosterone ELISA before and after exposure to chronic stress. An increase in mean urine corticosterone concentrations indicates an increase in physiological stress levels. There was no significant interaction of Housing Condition and Stress Exposure, *F*_(1, 40)_ = 0.70, *p* = 0.40, $\eta_{p}^{2}$, indicating that there was no significant difference in the increase in urine corticosterone levels between socially-housed and singly-housed mice. Dots represent individual data points in each group, bars represent to mean for each group. See Supplementary Materials for pairwise comparisons.

Mean urine corticosterone concentration (ng/mL) was the dependent measure (Figure 3), where greater urine corticosterone levels would indicate higher stress levels. Results showed a significant main effect of Immobilization with a large effect size, *F*_(1, 40)_ = 111.69, *p* < 0.000, $\eta_{p}^{2}$ = 0.84. There was not a significant main effect of Housing Condition on urine corticosterone levels, *F*_(1, 40)_ = 3.06, *p* = 0.09, $\eta_{p}^{2}$ = 0.14, and no significant interaction *F*_(1, 40)_ = 0.70, *p* = 0.40, $\eta_{p}^{2}$ =0.03. See Supplementary Materials for group summary.

Altogether, the significant main effects from the linear mixed effects models for the open field test and corticosterone ELISAs are sufficient to show that repeated immobilization was successful in inducing stress as measured both physiologically and behaviorally. (Supplemental pairwise comparisons, with Tukey's corrections, using the estimated marginal means in Table S1 are shown in Table S2).

### 3.2. Socially-Housed Mice Exhibited a Smaller Increase in High-Risk Decision-Making After Exposure to Chronic Stress Than Did Singly-Housed Mice

**Figure 4:**
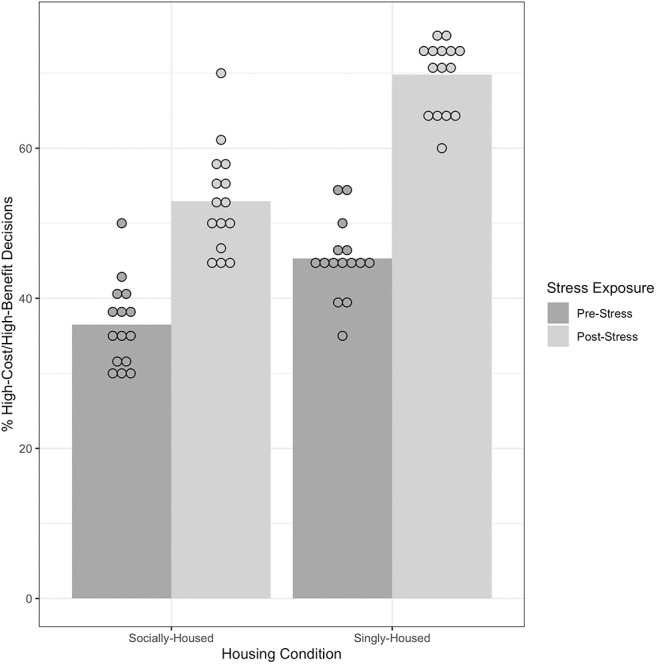
Performance of socially-housed (*n* = 15) and singly-housed mice (*n* = 15) on the Cost-Benefit Conflict task before and after exposure to chronic stress. An increase in % high-risk/high-reward decisions indicates an increase in preference for immediate payoffs (adaptive decision-making). There was a significant interaction of Housing Condition and Stress Exposure, *F*_(1, 28)_ = 9.56, *p* = 0.004, $\eta_{p}^{2}$ = 0.24, indicating that socially-housed mice showed a smaller increase in % high-risk/high-reward decisions than singly-housed mice after exposure to chronic stress. Dots represent individual data points in each group, bars represent to mean for each group. See Supplementary Materials for pairwise comparisons.

We ran a linear mixed effects model, with two fixed factors Immobilization and Housing Condition. Mouse was a random factor. Mean % of decisions in which mice chose the high-risk/high-reward alternative was the dependent measure. There was a significant main effect of Immobilization on % of high-risk/high-reward decisions with a large effect size, *F*_(1, 28)_ = 248.53, *p* < 0.000, $\eta_{p}^{2}$ = 0.89, indicating that, in general, mice chose the high-risk/high-reward alternatives at a significantly higher rate after exposure to chronic immobilization than before. There was also a significant main effect of Housing Condition on % of high-risk/high-reward decisions, *F*_(1, 28)_ = 69.84, *p* < 0.000, $\eta_{p}^{2}$ = 0.70, indicating that singly-housed mice chose the high-risk/high-reward alternatives at significantly higher rates than socially-housed mice. Finally, there was also a significant interaction between Immobilization and Housing Condition on the % of high-risk/high-reward decisions made by the mice, *F*_(1, 28)_ = 9.56, *p* = 0.004, $\eta_{p}^{2}$ = 0.24. This significant interaction indicates that the increase in high-risk/high-reward choices after chronic immobilization differed between the socially- and singly-housed animals. Figure 4 and the means summarized in Tables S1, S2 show that the increase in % of high-risk choices by the socially-housed animals was smaller than the increase for singly-housed animals.

## 4. Discussion

This study investigated the role of social isolation in exacerbating the effects of chronic stress on decision-making in mice. We hypothesized that mice would make more frequent high-risk/high-reward decisions after being chronically-stressed, and that this adaptive change in decision-making would be greater in mice that are housed singly than those housed in groups. Indeed, we found that, regardless of housing condition, mice showed an increased preference for the high-risk/high-reward alternative in the decision-making task after chronic stress exposure. This finding confirms the observations of Friedman et al. (2017). Furthermore, the current study shows that socially-housed mice exhibited a smaller increase in high-risk/high-reward preferences compared to singly-housed mice. Critically, we have demonstrated that the lack of social interaction may have a exacerbating influence on the effects of chronic stress.

In addition, the current study also confirms findings from previous research investigating chronic stress, social interaction, and cognition. In our study, mice were immediately assigned to singly-housed or socially-housed groups upon arrival of mice to our housing facilities. The mice were in these housing conditions for five days before pre-stress testing. Prior to the repeated immobilization protocol, we observed differences between the housing groups on both the corticosterone measure and the CBC results (Table S2). This finding confirms previous literature which suggested that social isolation can have stress-like influences on the behavior and physiology of rodents (Paolo et al., 1994; Nilsson et al., 1999; Zelikowsky et al., 2018). Our study extends this knowledge by showing that social isolation can significantly alter decision-making.

One aspect of the study which limits generalization of the results is that all the mice used in our study were adult male mice. We chose to use only male mice in our study to allow for comparison with previous studies that have investigated similar research questions such as Friedman et al. (2017), which also studied only male mice. However, studies do show that female mice exhibit different chronic stress responses than male mice (Franceschelli et al., 2014), including differential behavioral and physiological reactivity (Yamaura et al., 2013; Sachs et al., 2014; Karisetty et al., 2017). Additionally, a recent study by Dadomo et al. (2018) has suggested that female mice are more susceptible to nonsocial stressors compared to social stressors (such as isolation). In fact, social isolation may lead to opposite behavioral responses among male and female mice (Palanza et al., 2001). This further complicates our findings when attempting to generalize to female mice, since social interaction may influence stress responses differently in female mice than in male mice. We also used relatively young adult mice (about 8 weeks old), and certainly there are some age-related differences in behavioral and physiological responses to chronic stress among mice (Toth et al., 2008; Sadler and Bailey, 2016). Therefore, care must be taken when attempting to generalize the findings of the current study.

There are also certain limitations to the use of the Open Field Test as a measure of stress-sensitive anxiety-like behavior. Although the Open Field Test has been classically used as an indicator of stress and anxiety in rodents, this paradigm is sensitive to differences in *motor activity* in a novel arena. Thus, performance on an Open Field Test may well be influenced by a number of factors including the arousal level of mice at the time of testing and motor function, rather than just anxiety level. In the current study, only time spent in the center of the novel arena was recorded during the Open Field Test. Other open field measures, such as total distance traveled or time spent grooming in the open field, were not recorded. These additional measures are important in interpreting behavioral data in response to the repeated immobilization and social isolation. Notably, our secondary stress measure, urine corticosterone levels, did show that our immobilization protocol was successful in causing stress responses in both groups of mice.

Despite these limitations, the current study makes strong contributions to our understanding of how social isolation can amplify the effects of chronic stress on decision-making. Methodologically, results from the current study confirmed that our short, 7-day repeated immobilization protocol induced stress levels that led to measurable changes in both behavior and physiology. A 2-week period of immobilization is commonly used to induce chronic restraint stress in mice (Han et al., 2015; Friedman et al., 2017). However, mice can habituate quite quickly to a stressor. Thus, it is crucial to balance the time required to reliably induce measurable changes in stress levels against the potential for habituation. The current study used a 7-day period of repeated immobilization, which has been used to measure chronic stress effects in rodents previously (Li et al., 2013). We observed that stress levels among mice increased significantly after the 7-day immobilization protocol in both the Open Field Test and the confirmatory urine corticosterone assays. These results thus confirm that a shorter 7-day stress-induction protocol may also be effective in reliably inducing chronic restraint stress in mice.

Additionally, our study demonstrates that chronic stress can cause changes in decision-making and lead to preferences for high-risk/high-reward decisions over lower risk alternatives that yield a lower reward. This could be due to a mechanism wherein chronically stressful experiences may create a mindset (or cognitive bias) that favors high rewards. Indeed, in conditions of high stress or low environmental predictability, a bias toward riskier immediate awards may in fact be adaptive for an organism. There is considerable active interest in exploring the underlying neural mechanisms that produce this adaptive response for maximizing reward (Orsini et al., 2015). The mesocorticolimbic system, including the ventral tegmental area and its projections to the nucleus accumbens, amygdala, hippocampus, and medial prefrontal cortex (mPFC), is strongly implicated in goal-directed behaviors like decision-making (Ernst and Paulus, 2005; Venkatraman et al., 2009). Physiological manipulations such as chronic drug use have been shown to bias the system toward high-risk/high-reward choices (Kohno et al., 2014), and it is likely that chronic stress influences this system as well. For example, Cerqueira et al. (2007) found that chronic stress in rats induced selective atrophy in the PFC and reduced induction of long-term potentiation of the hippocampal-PFC pathways. More research is needed to better understand how molecular mechanisms (such as stress hormones) may modify these neural circuits. Moreover, studies can also directly investigate the adaptiveness of a high-risk/high-reward bias in stress environmental conditions using ecological methods.

Finally, we show that long-term lack of social interaction exaggerates the effects of chronic stress, and thus, conversely, this study offers a model for mitigating the effects of chronic stress on cognition. Much more investigation is needed to understand the mechanisms and the limits of this effect. For instance, chronic stress due to repeated immobilization may interact differently with social isolation than chronic stress due to social defeat (Golden et al., 2011), in which mice are repeatedly subjected to agonistic confrontations by a larger and more aggressive mouse leading to significant social stress. Since social defeat would arguably cause subsequent social interactions to be a negative affective experience for mice, social interaction may exacerbate, not mitigate, stress effects in mice that were chronically stressed through social defeat. Thus, while establishing a relationship between chronic stress and social isolation using one stress protocol, the current study also raises questions for future research about the influence of different types of stressors on this relationship.

Our findings offer a framework for human research into the effects of various stress-inducing experiences on cognition. In particular, results from mouse studies have been reliably extrapolated to studying conditions such as anxiety or depression in humans (Cryan and Holmes, 2005). In fact, unpredictable chronic mild stress (UCMS), a well-established chronic stress protocol, has often been used in mouse studies to screen antidepressant drug candidates (Nollet et al., 2013), underlining the value of mouse models in translational research into psychological disorders. The interrelationship between social interaction, chronic stress, and decision-making has significant implications for society, especially for professionals in high-stress jobs that often have to make decisions with wide-ranging consequences regularly, such as emergency medical professionals or finance consultants. While care should be taken whenever generalizing specific findings from animal studies to human behavior, the current study provides a broad framework suggesting that the absence of healthy social interaction may play a crucial role in exacerbating the adaptive changes in decision-making that may be caused by chronic stress among these individuals.

One major insight from the current study is that chronic stress experience may cause changes in decision-making, which provides a new framework for understanding the factors that influence human cognition. Future research could explore what other stress-inducing experiences cause changes in decision-making. Are those changes domain-specific or domain-general? For example, does chronic social stress cause changes selectively to social cognition, or all cognitive processes generally? Another interesting avenue for research could be exploring systemic “minority stress” effects which have been observed among many marginalized population groups (Meyer, 1995). People living in poverty, LGBTQ+ individuals, and ethnic and racial minorities are some groups that experience both covert and overt discrimination in daily life, which can be thought of as chronic stress (Evans and Kim, 2013). A growing body of health-related research suggests that this chronic marginalization stress may negatively impact health and well-being in much the same way as more commonly studied forms of chronic stress (Brunner, 1997; Lehmiller, 2012; Borders et al., 2015; Wheeler et al., 2018). Findings from the current study support arguments that observed increases in preferences for immediate payoffs among these groups, often characterized as “irresponsible” behavior inherent among marginalized groups, may in fact be an adaptive response to chronic stress. Furthermore, the current study offers evidence for the potential of social interaction to mitigate these changes in decision-making caused by chronic stress. With further research, findings from the current study may inform public health policies that combat chronic stress effects among marginalized populations.

The current study was the first to simultaneously examine the effects of chronic stress and social isolation on decision-making using a mouse model. We demonstrated that mice showed an increased preference for high-risk/high-reward alternatives after exposure to chronic stress regardless of housing condition. More importantly, socially-housed mice showed a smaller increase in proportion of high-risk/high-reward decisions after exposure to chronic stress when compared to singly-housed mice. Therefore, we found that chronic stress leads to an increase in high-risk decision-making in mice, an effect which is further exacerbated by social isolation. Through the current study, we offer a model for influencing chronic stress effects on cognition among individuals. Our findings suggest that an increase in high-risk decision-making may be caused by chronic stress, and that social interaction may play a crucial role in mitigating these effects.

## Data Availability Statement

The datasets analyzed for this study, as well as the code used for analysis, can be found in the repository named Arish Mudra Rakshasa at https://github.com/michelletytong/MudraRakshasa.

## Ethics Statement

The animal study was reviewed and approved by Earlham College Institutional Animal Care and Use Committee (IACUC).

## Author Contributions

AM conceived the study, carried out the experiments, and wrote the paper. MT aided in experimental design, data analysis, and manuscript preparation.

## Conflict of Interest

The authors declare that the research was conducted in the absence of any commercial or financial relationships that could be construed as a potential conflict of interest.
