# Supplementary material for: Making “Good” Choices: Social Isolation in Mice Exacerbates the Effects of Chronic Stress on Decision Making
Source: Front Behav Neurosci. 2020 May 25;14:81. doi: 10.3389/fnbeh.2020.00081 (PMC7261864; doi:10.3389/fnbeh.2020.00081)
Supplement: Supplementary file 1 [file Data_Sheet_1.PDF]

Supplementary Material

1 SUPPLEMENTARY DATA

Group summary statistics and pairwise comparisons for our linear mixed effects models are presented here.

Table S1. Estimated Marginal Means and Standard Deviations for each group for all 3 dependent variables

DV	Condition	Stress Exposure	Mean	Std Dev
Open Field	Socially-housed	Pre-stress	26.5 sec	1.02
	Socially-housed	Post-stress	20.3 sec	1.02
	Singly-housed	Pre-stress	26.1 sec	1.02
	Singly-housed	Post-stress	15.6 sec	1.02
ELISA	Socially-housed	Pre-stress	3.19 (ng/mL)	0.486
	Socially-housed	Post-stress	8.44 (ng/mL)	0.486
	Singly-housed	Pre-stress	4.38 (ng/mL)	0.486
	Singly-housed	Post-stress	8.86 (ng/mL)	0.486
CBC	Socially-housed	Pre-stress	36.5 (%)	1.47
	Socially-housed	Post-stress	52.9 (%)	1.47
	Singly-housed	Pre-stress	45.3 (%)	1.47
	Singly-housed	Post-stress	69.8 (%)	1.47

Table S2. Pairwise comparisons for each

DV	Group	Comparison	t(df)	p-value
Open Field	Socially-housed	Pre-stress - Post-stress	$t(32.1) = 7.095$	0.000
	Singly-housed	Pre-Stress - Post-stress	$t(32.1) = 12.11$	0.000
	Pre-Stress	Socially - Singly	$t(45.8) = 0.223$	1.000
	Post-Stress	Socially - Singly	$t(45.8) = 3.246$	0.013
ELISA	Socially-housed	Pre-stress - Post-stress	$t(22.2) = -7.651$	0.000
	Singly-housed	Pre-stress - Post-stress	$t(22.2) = -6.528$	0.000
	Pre-stress	Socially - Singly	$t(44.4) = -1.735$	0.318
	Post-stress	Socially - Singly	$t(44.4) = -0.611$	0.928
CBC	Socially-housed	Pre-stress - Post-stress	$t(32.1) = -8.658$	0.000
	Singly-housed	Pre-stress - Post-stress	$t(32.1) = -12.882$	0.000
	Pre-stress	Socially - Singly	$t(62.5) = -4.243$	0.000
	Post-stress	Socially - Singly	$t(62.5) = -8.096$	0.000
